# Apo A1/Apo B ratio and acute coronary syndrome among peritoneal dialysis patients

**DOI:** 10.1080/0886022X.2021.1918556

**Published:** 2021-04-29

**Authors:** Tianlei Chen, Min Yang

**Affiliations:** Department of Nephrology, The First People’s Hospital of Changzhou, The Third Affiliated Hospital of Soochow University, Changzhou, PR China

**Keywords:** Apoprotein A1, apoprotein B, acute coronary syndrome, continuous ambulatory peritoneal dialysis

## Abstract

**Background:**

Acute coronary syndrome (ACS) is prevalent in continuous ambulatory peritoneal dialysis (CAPD) patients. However, the association between the apoprotein profile and ACS is not well known. Therefore, we aimed to investigate the relationship between apoproteins and ACS in CAPD patients.

**Methods:**

Eighty-one CAPD patients were included in this retrospective study. The primary endpoint was ACS. Predictors were baseline apoprotein levels, particularly the ratio of apoprotein A1 (Apo A1)/apoprotein B (Apo B). Cox regression was used to determine the relationship between Apo A1/Apo B and ACS.

**Results:**

During follow-up, 34 (41.98%) CAPD patients experienced an ACS. ACS patients had higher levels of total cholesterol (*p* = 0.03), low-density lipoprotein cholesterol (LDL-C) (*p* = 0.04), C-reactive protein (*p* = 0.01), and Apo B (*p* < 0.01). However, hemoglobin (*p* = 0.01) and Apo A1/Apo B (*p* < 0.01) were lower in the ACS group than the non-ACS group. Patients with Apo A1/Apo *B* ≥ 1.105 experienced fewer ACS compared with those with Apo A1/Apo *B* < 1.105 (33.33% *vs.* 75.56%, *p* = 0.03). In Cox regression, Apo A1/Apo B (RR, 0.06; 95% CI, 0.00-0.77; *p* = 0.03) was independently associated with ACS.

**Conclusions:**

Apo A1/Apo B was strongly associated with ACS and may be considered as a predictor of future ACS in CAPD patients.

## Introduction

More than 10% of people worldwide have chronic kidney disease (CKD) [1]. As renal function declines, the incidence of cardiovascular disease (CVD) in patients increases significantly. CKD is an important predictor of adverse cardiovascular events [2,3]. Although these patients have the potential for many complications, their risk of developing acute coronary syndrome (ACS) becomes exceptionally high due to accelerated vascular disease and the presence of other comorbidities. In addition to a higher incidence of ACS than in the general population, patients with CKD also have worse ACS related outcomes [4–6]. Mortality rates of 59% and 90% at 1 and 5 years have been reported in dialysis patients with CKD stage 5 after concomitant ACS, respectively, which are much higher than those in ACS patients without CKD [7]. Therefore, most scholars believe that CKD is an independent factor of ACS. The occurrence and development of CKD and ACS affect each other, exacerbating the adverse outcome of patients.

Hyperlipidemia is a recognized risk factor for CVD in the general population [8–10]. Dyslipidemia is the most important risk factor for coronary atherosclerosis, which in turn underlies ACS lesions. However, in CKD patients, the relationship becomes more complex and may even be contradictory [11]. CKD seems to weaken the classical relationship between abnormal lipid metabolism and CVD [12]. At this time, the apoprotein A1 (Apo A1)/apoprotein B (Apo B) ratio provides a better summary of the burden of dyslipidemia than conventional lipids and lipoproteins. Previous studies [13] have confirmed that Apo A1/Apo B is closely related to CVD risk in maintenance hemodialysis patients. However, the relationship between apolipoprotein profile and ACS in continuous ambulatory peritoneal dialysis (CAPD) patients is poorly studied. Therefore, this study aimed to investigate the relationship between Apo A1/Apo B and ACS in CAPD patients.

## Patients and methods

### Study subjects

**Figure 1.**
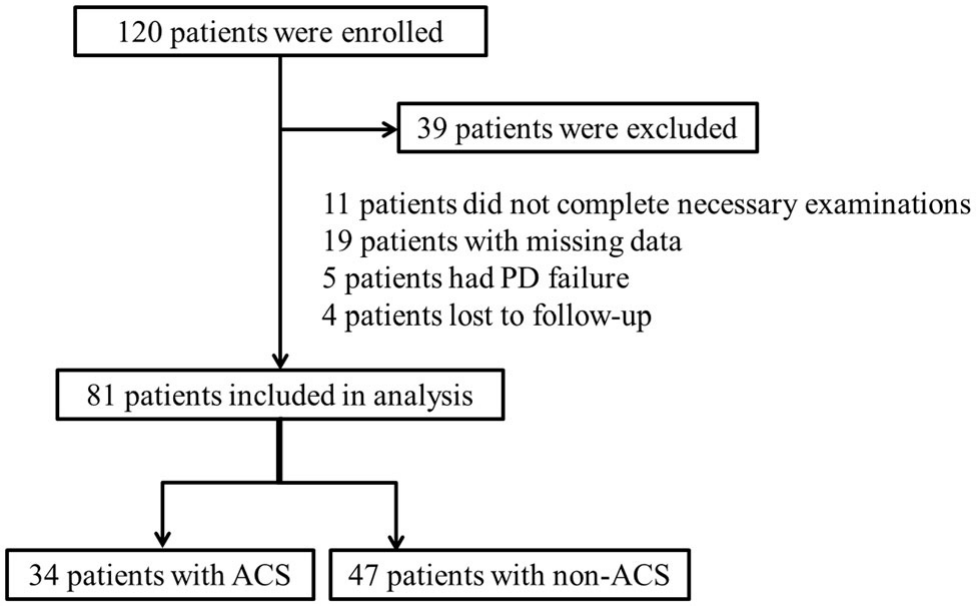
Flowchart of patient selection.

As shown in Figure 1, 81 of 120 patients with regular CAPD who were hospitalized in the Nephrology Department of our hospital from February 2014 to December 2017 were finally enrolled. Inclusion criteria included age greater than 18 years, diagnosis of uremia, and regular peritoneal dialysis greater than or equal to 3 months, and the specific peritoneal dialysis protocol had to be CAPD mode. At the same time, patients who could not cooperate to complete the relevant index examination (*n* = 11), missing data (*n* = 19), had peritoneal dialysis failure during follow-up (*n* = 5), and lost to follow-up (*n* = 4) were excluded. The study was carried out following the Declaration of Helsinki and approved by the Medical Ethics Committee of the Third Affiliated Hospital of Soochow University (Number: 2014-24).

### Data collection

From enrollment, data on age, gender, body mass index (BMI), smoking, hypertension, diabetes, history of CVD, primary disease of CKD, use of statins, residual glomerular filtration rate (GRF), serum creatinine, urea nitrogen, lipid profile, albumin, hemoglobin, intact parathyroid hormone (iPTH), serum calcium, serum phosphate and C-reactive protein (CRP) were collected from case records. The above laboratory parameters were measured by the central laboratory in an automatic system and a standardized method. The Apo A1/Apo B ratio was obtained by dividing the absolute value of Apo A1 by the absolute value of Apo B.

### Follow-up of CAPD patients

The enrolled CAPD patients were followed up by outpatient, inpatient, telephone, or home visited. The primary endpoint of this study was the occurrence of ACS during follow-up. ACS includes acute ST-segment elevation myocardial infarction (STEMI), acute non-ST-segment elevation myocardial infarction, and unstable angina pectoris, which were diagnosed by clinical symptoms, dynamic electrocardiogram changes, and serum myocardial injury markers.

### Statistical analysis

Statistical analysis was performed using SPSS software (*v. 19.0*, IBM Corp., Armonk, NY). Continuous variables were expressed as mean ± standard deviation, and categorical variables were presented as frequency and percentage. The cutoff values of Apo A1/Apo B were analyzed using ROC curves. Survival curves according to Apo A1/Apo B were determined by K-M survival analysis. Cox regression analysis was performed to evaluate the association between Apo A1/Apo B and ACS. A 2-tailed *p* < 0.05 was considered statistically significant.

## Results

### Comparison of demographic and clinical characteristics between ACS and non-ACS groups

Eighty-one CAPD patients were included in this retrospective study. During follow-up, a total of 34 (41.98%) patients experienced ACS. Patients were divided into two groups according to the presence or absence of ACS. No statistical differences were found in age (*p* = 0.76), sex (*p* = 0.50), BMI (*p* = 0.86), smoking (*p* = 0.38), hypertension (*p* = 0.81), diabetes (*p* = 0.79), history of CVD (*p* = 0.51), etiology of CKD (*p* > 0.05), use of statins (*p* = 0.44), Follow-up duration (*p* = 0.44), total *k_t_/v_urea_* (*p* = 0.07), residual GFR (*p* = 0.55), urea nitrogen (*p* = 0.17), serum creatinine (*p* = 0.43), triglycerides (*p* = 0.11), high-density lipoprotein cholesterol (HDL-C) (*p* = 0.22), albumin (*p* = 0.65), iPTH (*p* = 0.97), serum calcium (*p* = 0.87), serum phosphate (*p* = 0.80), and Apo A1 (*p* = 0.09) between the CVD and non-CVD groups. Moreover, as showed in Table 1, patients in the ACS group had higher levels of total cholesterol (*p* = 0.03), low-density lipoprotein cholesterol (LDL-C) (*p* = 0.04), CRP (*p* = 0.01), and Apo B (*p* < 0.01). However, hemoglobin (*p* = 0.01) and Apo A1/Apo B (*p* < 0.01) were significantly lower in the ACS group than in the non-ACS group.

**Table 1.** Clinical data of 81 peritoneal dialysis patients.

Variables	Non-ACS (*n* = 47)	ACS (*n* = 34)	*p* Value
Age	50.47 ± 12.54	49.74 ± 8.90	0.76
Female	18	16	0.50
BMI, kg/m^2^	21.60 ± 2.42	21.48 ± 3.12	0.86
Smoking	10	4	0.38
Hypertension	28	22	0.81
Diabetes	12	7	0.79
History of CVD	5	6	0.51
CKD etiology			
Chronic glomerulonephritis	33	23	0.81
Diabetic nephropathy	9	8	0.78
Hypertensive nephropathy	1	3	0.30
Other	4	0	0.14
Use of Statins	33	27	0.44
Follow-up duration	24.91 ± 16.04	22.70 ± 9.13	0.44
Total *k_t_/v_urea_*	1.81 ± 0.53	2.05 ± 0.54	0.07
Residual GFR, ml/min/1.73m^2^	2.44 ± 1.95	2.75 ± 2.49	0.55
Urea nitrogen, mmol/L	17.86 ± 5.01	19.51 ± 5.66	0.17
Serum creatinine, umol/L	810.89 ± 195.76	773.62 ± 226.48	0.43
Triacylglycerol, mmol/L	2.62 ± 1.50	3.21 ± 1.84	0.11
Total cholesterol, mmol/L	3.98 ± 1.00	4.52 ± 1.09	0.03
LDL-C, mmol/L	2.16 ± 0.68	2.50 ± 0.76	0.04
HDL-C, mmol/L	0.96 ± 0.28	0.90 ± 0.18	0.22
Albumin, g/L	30.41 ± 4.78	29.90 ± 5.36	0.65
Hemoglobin, g/L	100.93 ± 13.70	92.82 ± 13.25	0.01
iPTH, ng/L	288.85 ± 150.06	289.97 ± 117.55	0.97
Serum calcium, mmol/L	2.10 ± 0.23	2.09 ± 0.22	0.87
Serum phosphate, mmol/L	1.62 ± 0.30	1.60 ± 0.27	0.80
C-reactive protein, mg/L	6.37 ± 1.98	7.94 ± 3.05	0.01
Apo A1, mg/dL	105.28 ± 14.17	100.03 ± 12.40	0.09
Apo B, mg/dL	86.11 ± 16.78	99.32 ± 14.63	< 0.01
Apo A1 / Apo B	1.26 ± 0.28	1.02 ± 0.16	< 0.01

ACS: acute coronary syndrome; BMI: body mass index; CVD: cardiovascular diseases; CKD: chronic kidney disease; GFR: residual glomerular filtration rate; LDL-C: low-density lipoprotein cholesterol; HDL-C: high-density lipoprotein cholesterol; iPTH: Intact parathyroid hormone; Apo A1: apolipoprotein A1; Apo B: apolipoprotein B.

### Apo A1/Apo B and ACS

**Figure 2.**
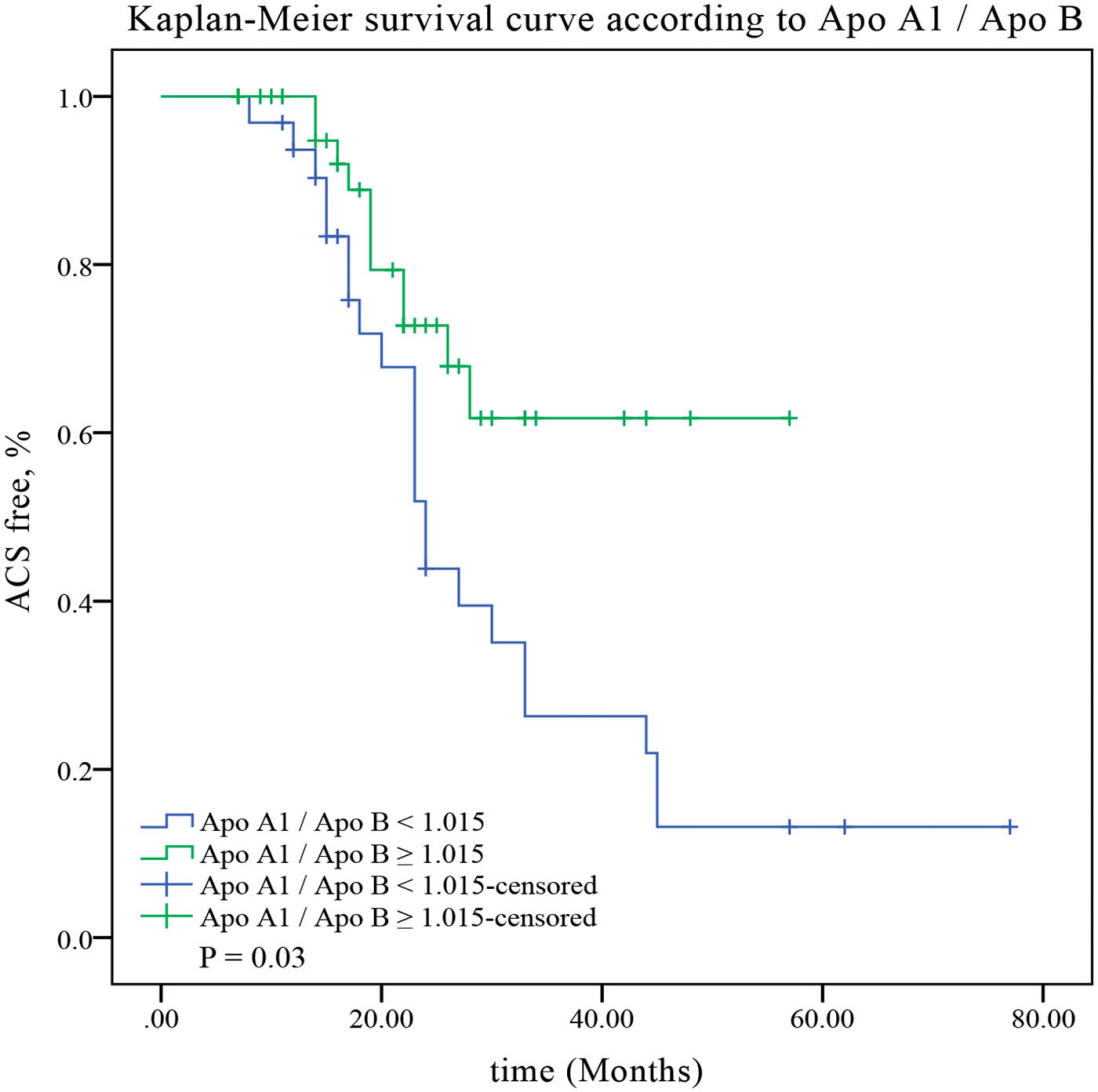
ACS free survival curves according to Apo A1/Apo B. ACS: acute coronary syndrome; Apo A1: apolipoprotein A1; Apo B: apolipoprotein B.

**Figure 3.**
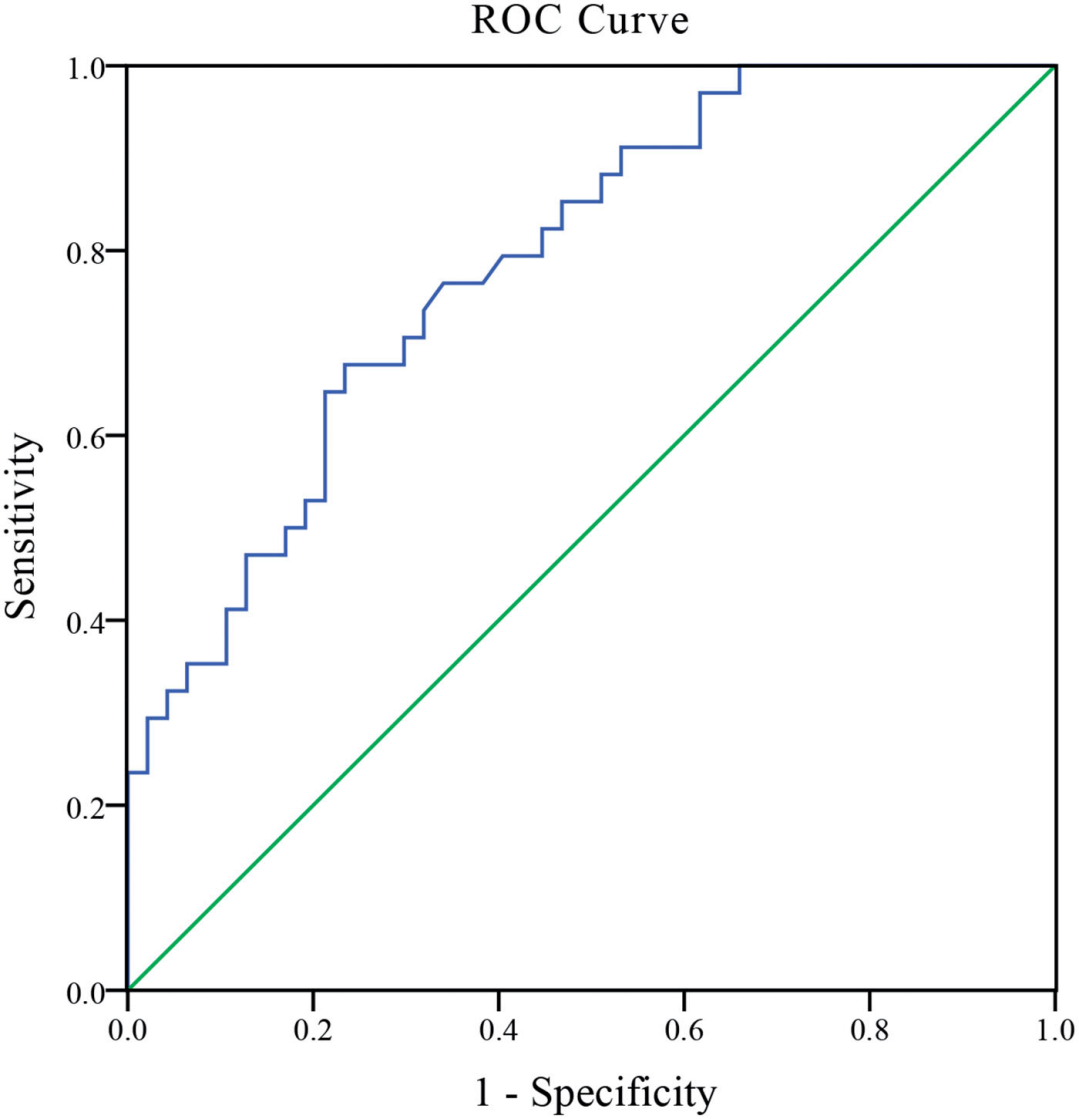
ROC curves analysis for Apo A1/Apo B. Apo A1: apolipoprotein A1; Apo B: apolipoprotein B.

In the second step, the optimal cutoff value of Apo A1/Apo B was calculated using ROC curve analysis. As shown in Figure 3, the optimal cutoff for Apo A1/Apo B was 1.105. Subsequently, we found that patients with Apo A1/Apo *B* ≥ 1.105 experienced fewer ACS during follow-up compared with those with Apo A1/Apo *B* < 1.105 (Figure 2; 33.33% *vs.* 75.56%, *p* = 0.03).

### Apo A1/Apo B was associated with ACS

According to Figure 3, the area under the ROC curve for Apo A1/Apo B was 0.79 (95% CI: 0.69–0.88, *p* < 0.01). In Cox regression (Table 2), Apo A1/Apo B (RR, 0.06; 95% CI: 0.00–0.77; *p* = 0.03) was independently associated with ACS in CAPD patients, Even after adjustment for age (RR, 0.98; 95% CI: 0.94–1.02; *p* = 0.35), history of CVD (RR, 0.97; 95% CI: 0.30–3.16; *p* = 0.96), diabetes (RR, 0.56; 95% CI: 0.18–1.74; *p* = 0.32), smoking (RR, 0.77; 95% CI: 0.23–2.62; *p* = 0.67), triglycerides (RR, 0.90; 95% CI: 0.70–1.16; *p* = 0.42), total cholesterol (RR, 1.29; 95% CI: 0.78–2.15; *p* = 0.32), CRP (RR, 0.93; 95% CI: 0.78–1.12; *p* = 0.46), hemoglobin (RR, 0.99; 95% CI: 0.95–1.03; *p* = 0.53), albumin (RR, 1.08; 95% CI: 1.00–1.18; *p* = 0.07) and Apo B (RR, 1.00; 95% CI: 0.97–1.03; *p* = 0.93).

**Table 2.** Cox regression analysis of ACS in peritoneal dialysis patients.

	RR	95% CI	*p* Value
age	0.98	0.94–1.02	0.35
History of CVD	0.97	0.30–3.16	0.96
Diabetes	0.56	0.18–1.74	0.32
Smoking	0.77	0.23–2.62	0.67
Triacylglycerol, mmol/L	0.90	0.70–1.16	0.42
Total cholesterol, mmol/L	1.29	0.78–2.15	0.32
C-reactive protein, mg/L	0.93	0.78–1.12	0.46
Hemoglobin, g/L	0.99	0.95–1.03	0.53
Albumin, g/L	1.08	1.00–1.18	0.07
Apo B, mg/dL	1.00	0.97–1.03	0.93
Apo A1 / Apo B	0.06	0.00–0.77	0.03

ACS: acute coronary syndrome; CVD: cardiovascular diseases; LDL-C: low-density lipoprotein cholesterol; HDL-C: high-density lipoprotein cholesterol; Apo B: apolipoprotein B; Apo A1: apolipoprotein A1; RR: relative risk.

Finally, bivariate correlation analysis was used to evaluate the association between apoproteins and CRP. As a result, no correlation was found between CRP and Apo A1 (*r* = 0.03, *p* = 0.77), Apo B (*r* = 0.20, *p* = 0.09), or Apo A1/Apo B (*r* = −0.20, *p* = 0.09).

## Discussion

Our study demonstrated the relationship between the Apo A1/Apo B ratio and ACS in CAPD patients. The association remained significant even after adjustment for traditional CVD risk factors such as age, prior CVD history, diabetes, smoking, triglycerides, total cholesterol, CRP, albumin, hemoglobin, and Apo B. Moreover, Apo A1/Apo B may be used as a predictor of the occurrence of future ACS.

Hyperlipidemia is a known risk factor for CVD in non-CKD patients [9,14]. CKD appears to weaken the classical relationship between the lipid and CVD. Elevated very-low-density lipoprotein and low HDL-C have been shown to increase coronary heart disease risk in CKD patients [15,16]. However, no significant association was found between LDL-C and cardiovascular outcomes. Moreover, some earlier studies [11,17,18] have failed to confirm an association between HDL-C, LDL-C, or triglyceride levels and CVD mortality. Similarly, our study indicated that total cholesterol and LDL-C were significantly higher in the ACS group than in the non-ACS group, but these two lipid parameters were not correlated with ACS. The study by Chang et al. [19] even came to the opposite conclusion that an increased triglyceride/HDL-C ratio was associated with reduced CVD mortality in hemodialysis patients. Therefore, the association between dyslipidemia and CVD in the CKD population is very complicated.

Apo A1 is the main component of high-density lipoprotein (HDL) and is closely related to CVD. A study [20] of 299 patients with STEMI found that high baseline Apo A1 levels were associated with reduced risk of STEMI. Apo B is present on the surface of low-density lipoprotein (LDL), and cellular recognition and uptake of LDL are mainly achieved by recognition of Apo B. Higher levels of Apo B can increase the incidence of coronary heart disease, even if LDL levels are normal. Moreover, Apo B was significantly associated with CVD related mortality [13]. Our study suggested that Apo A1 levels were higher in the non-ACS group than in the ACS group, while Apo B showed the opposite. Unfortunately, we did not conclude that Apo A1 or Apo B was associated with ACS.

The Apo A1/Apo B ratio provides a better summary of the burden of dyslipidemia than conventional lipids and lipoproteins. Some studies have confirmed that the Apo B/Apo A1 ratio was superior to LDL-C and HDL-C in predicting cardiovascular events. Bodde et al. [20] found that elevated Apo B/Apo A1 ratio was associated with an increased risk of STEMI, but not LDL-C, after adjustment for age, sex, and statin therapy. In maintenance hemodialysis patients, each SD increase in the Apo B/Apo A1 ratio increased all-cause mortality or CVD-related mortality by 16% [13]. In our study, similar results were found, adjusted for traditional CVD influencing factors, Apo A1/Apo B and ACS were independently associated. Furthermore, the area under the ROC curve for Apo A1/Apo B was 0.79 (95% CI: 0.69–0.88, *p* < 0.01). This suggests that Apo A1/Apo B may be well used as a predictor for the future occurrence of ACS in CAPD patients.

Andronesi et al. [21] found that advanced age (older than 55 years), smoking, and low iPTH (iPTH less than 150 pg/ml) were associated with a high risk of coronary heart disease in peritoneal dialysis patient without diabetic nephropathy. This is different from the conclusion of the present study. The discrepancy was due to population differences between the two studies. Our study population included patients with diabetic nephropathy, while patients in the ACS group were characterized by younger age (Mean age was 49.74 ± 8.90 years), lower rates of smoking (11.76%), and a smaller proportion of patients with low iTPH (The proportion of patients with iPTH less than 150 pg/mL was 11.76%). Moreover, meta-analyses [22] have confirmed that patients with low BMI after ACS have higher mortality rates than patients with normal BMI, overweight, obesity, and severe obesity. However, considering that there was no statistical difference in BMI between the ACS group and non-ACS group, and almost all study populations had normal BMI values in this study, we did not make a further adjustment for BMI.

Inflammation may affect Apo A1 metabolism to some extent in patients with renal failure, and CRP was found to be significantly higher in the CVD group than in the non-CVD group [23]. Our study also found that CRP was significantly higher in the ACS group than in the non-ACS group. However, no correlation was found between CRP and Apo A1, Apo B, and Apo A1/Apo B in the binary correlation analysis. At the same time, no correlation was found between CRP, albumin, and ACS in CAPD patients in the Cox regression analysis of this study. Therefore, apolipoproteins do not simply mediate the occurrence of ACS through an inflammatory state, and the specific mechanism requires further investigation.

Our study has some limitations. This study was a retrospective small sample study, and the conclusion needs to be confirmed by prospective large sample studies in the future. Secondly, due to the voluntary principle, the enrolled patients pay more attention to their physical health than the non-enrolled patients, so the research population may have a certain degree of selection bias, which cannot well represent the general CAPD patients. Thirdly, some characteristics, such as low BMI and low incidence of diabetes/diabetic nephropathy, were unique to this study population, i.e. Asian CKD patients. This limits the generalizability of the data. Finally, it was unclear how dyslipidemia affected the development of ACS.

## Conclusions

In conclusion, we found that the Apo A1/Apo B ratio was independently associated with ACS in CAPD patients. Our study suggested that the ratio of Apo A1/Apo B may be considered as a predictor of future ACS risk compared with traditional lipid measures. The role of apolipoproteins in predicting ACS in CKD patients and their specific mechanisms require further investigation.
